# Tear proteomics analysis of patient suffered from delayed mustard gas keratopathy

**DOI:** 10.1186/s12953-022-00195-1

**Published:** 2022-08-10

**Authors:** Shahram Parvin, Alireza Shahriary, Hossein Aghamollaei, B. Fatemeh Nobakht M. Gh, Hasan Bagheri, Mostafa Ghanei, Seyed-Hashem Daryabari, Khosrow Jadidi, Masoud Arabfard

**Affiliations:** 1 Chemical Injuries Research Center, Systems Biology and Poisonings Institute, Baqiyatallah University of Medical Sciences, Tehran, Iran; 2 Education Office, Pasture Institute of Iran, Tehran, Iran

**Keywords:** Proteomics, Sulfur mustard, Ocular complications, Tear

## Abstract

Understanding the molecular and cellular mechanisms involved in the pathogenesis of ocular injured induced by mustard gas can help better identify complications and discover appropriate therapies. This study aimed to analyze the proteomics of tears of chemical warfare victims with mustard gas ocular injuries and compare it with healthy individuals. In this case-control research, 10 mustard gas victims with long-term ocular difficulties (Chronic) were included in the patient group, while 10 healthy persons who were age and sex matched to the patients were included in the control group. Schirmer strips were used to collect the tears of the participants. Proteomics experiments were performed using the high-efficiency TMT10X method to evaluate the tear protein profile, and statistical bioinformatics methods were used to identify the differently expressed proteins. 24 proteins had different expressions between the two groups. Among these 24 proteins, 8 proteins had increased expression in veterans’ tears, while the remaining 16 proteins had decreased expression. Reactome pathways were used to look at proteins with various expressions, and 13 proteins were found to be engaged in the immune system, 9 of which were effective in the innate immune system, and 5 proteins were effective in the complement cascade. Ocular mustard gas exposure may cause a compromised immune system on the eye’s surface, exposing the cornea to external and endogenous infections, and eventually causing corneal opacity and reduced vision.

## Background

Sulfur Mustard gas with the chemical formula of bis (2-chlorethyl) sulfide is a chemical agent with alkylating and blistering effects that was used for the first time in World War I. It was widely used by Iraqi forces in the Iran-Iraq war, and thousands of civilians and militaries were affected by the dangerous effects of this biological weapon [1]. Because of its fat solubility, mustard gas is readily absorbed through the skin and combines with the aqueous environment of cells, producing hydrochloric acid. Furthermore, it causes alkylation of intracellular proteins and enzymes as well as DNA damage, causing cell function to be disrupted and eventually cell death. Oxidative stress and inflammatory pathways are two other recognized changed processes [2, 3]. Considering its ease of production and storage, high toxicity, and lack of definitive treatment and antidote, sulfur mustard is one of the most easily available and inexpensive Weapons of mass destruction, therefore its use was banned by the Chemical Weapons Convention (CWC) and the Organization for the Prohibition of Chemical Weapons (OPCW). However, there is still a risk of it being used by terrorist groups [4–6]. About 63,000 approved chemical veterans are now battling the consequences of this gas in Iran, according to the General Director of the Iranian Chemical Injuries of Martyrs and Victims Affairs Foundation. The most vulnerable organ to mustard gas is the eye, which may be impacted at quantities 10 times lower than those needed to induce respiratory or cutaneous damage [6]. Even 30 years after their first exposure to sulfur mustard, 65% of veterans suffered from ocular issues, according to a research done by Balali-Mood and his colleagues [4]. The ocular complications of mustard gas are divided into two categories: acute and delayed complications. Eye pain, foreign body feeling, burning, anterior uveitis, conjunctivitis, photophobia, and temporary blindness are some of the acute consequences. Chronic inflammatory processes, sulfur mustard metabolites, and autoimmune responses might cause delayed consequences after an asymptomatic period. The most important delayed complications include neovascularization, corneal haze and scarring, corneal dystrophy, limbal stem cells defect, corneal thinning, limbus ischemia and lipid and amyloid deposition in the cornea [7–11].

Currently no definitive treatment exists for ocular complications of sulfur mustard. At the first stage of injuries, some medications including washing the eyes with plenty of water or normal saline, using sodium bicarbonate, dichloramine, artificial tears, topical antibiotics, corticosteroids, and midriatic agents can be used [8, 12]. Artificial tears, anti-inflammatory therapies, immunomodulatory pharmaceuticals, human blood derivatives, amniotic membrane transplantation, tarsoraphy, stem cell transplantation, and corneal transplantation are some of the pharmacological and surgical treatments for delayed problems [8, 13]. Understanding the molecular and cellular mechanisms involved in the pathogenesis of mustard gas keratopathy aids in identifying complications and discovering appropriate therapies. One of the ways to better understand the pathogenesis of diseases is to study the proteome of the organs involved. Proteomics is a studies that can detect protein changes in different tissues. Protein studies have been previously performed using antibodies and methods, such as Western blotting and ELISA that allowed for the examination of a limited number of proteins that were abundantly present in the sample. However, due to technological advancements and the advent of mass spectrometry, it is now feasible to analyze a wide range of proteins, even those with low abundance in varied samples. The most recent mass spectrometry technology is the iTRAQ, which is the most accurate in detecting proteins even with a small amount [14], and was used in this study to better evaluate the tear proteome. Moreover, in this study, we used the latest Tandem Mass tag (TMT) method, known as TMT10X, to better and more accurately identify proteins compared to earlier methods using gel or antibody assays [15]. Numerous proteomics research on various illnesses have been conducted in the recent decade, leading to a better knowledge of disease pathophysiology and the development of novel treatment methods [16]. Proteomics may also aid in the accurate diagnosis of illnesses, the prediction of disease outcomes, and the monitoring of patients. This study aimed to analyze the proteome of tear in warfare veterans with sulfur mustard ocular complications and compare it with healthy individuals, using the proteomics approach. Understanding the changes in the tear proteome of these patients may help better understand the pathogenesis of mustard gas ocular complications and be a prelude to devising more effective therapeutic approaches for these patients.

## Materials and methods

### Patients and controls

The study was approved by the Institutional Review Board and the Ethical Committee of Baqiyatallah University of Medical Sciences (code: IR.BMSU.REC.1395.381). The consent form was signed by all of the individuals that were recruited. Ten patients and ten controls who were matched in sex and age were enrolled in the research after completing the questionnaire and providing informed permission. Patients required to have medical records and their delayed mustard injuries had to be validated by specialist ophthalmologists to be included in the study. Besides, all moderate to severe confirmed DMGK patient with ischemia in the conjunctiva and limbus, vascular penetration to the corneal periphery, with or without opacity, corneal thinning and melting. All the subjects were adult and non-smokers and aged between 45 and 60 years. Furthermore, history of previous eye surgery, systemic disease including diabetes, blood pressure, chronic kidney disease, current eye infection, or diseases such as glaucoma, taken corticosteroids or other specific drugs for 2 weeks, seasonal allergies in the last 6 months, and treated with antibiotics for 2 weeks were excluded. Tear sampling was done by Schirmer strip (ERC, Turkey). Tears were collected without the use of any anesthetics. For 5 minutes, Schirmer paper was put at the outside third of the lower eyelid’s margin (below the fornix). The paper was then removed and put in the nuclease-free tube before being transported to the − 80 freezer.

### Sample preparation

For protein extraction, Schirmer strips were cut to the 2*2 mm and transferred to the 1.5 ml sterile micro tube. 800ul of 1% sodium deoxycholate in 100 mM NH4HCO3 was added to the samples and vortexed vigorously, the samples were centrifuged at 5000 RPM. The protein solution was lyophilized and wrapped in the cap using parafilm.

Protein concentrations were determined using BCA assay (Thermo Scientific, USA) as per manufacturer’s instruction. Cysteine disulphide bonds in the proteins were reduced with 10 mM DTT at 37 °C for 1 h, and then alkylated with 20 mM IAA for 45 min in dark at room temperature. Remaining IAA in the samples were quenched with 10 mM DTT for 15 min in the dark at room temperature. Samples were proteolyzed with Lys-C (100:1 protein to enzyme ratio) overnight at 28 °C, then digested with trypsin (100:1 protein to enzyme ratio) for 6 hours at 37 °C. The sample was desalted using a solid phase extraction disk containing Stage tips and Styrene Divinyl Benzene after the pH was adjusted to roughly 3 with a final concentration of around 1% TFA (Empore SDB-RPS 47 mm extraction disk, SUPLCO). Peptides were bonded to stage tips, washed with 0.2% TFA, and then eluted with 80% acetonitrile: 5% ammonium hydroxide. The peptides were vacuum centrifuged before being reconstituted in 200 mM HEPES pH 8.8 and measured using the Pierce quantitative colorimetric peptide assay (Thermo Scientific, USA).

### Sample labelling

TMT reagent labelling of peptides was performed as per manufacturer’s (Thermo Scientific, USA) instructions. Each TMT label vial was filled with anhydrous acetonitrile, vortexing for 5 minutes, and centrifugation. One of the individual TMT labels was applied to aliquots of individual peptide samples (total of ten labels). Labeling took place at room temperature for 1 hour, with some vortexing in between. TMT labelling scheme for each sample is listed in Table 1.

**Table 1:** The TMT labelling scheme used for each individual tear sample

TMT Labels (tear samples)	TMT-1	TMT-2
**126**	**T2(Control)**	**T2(Control)**
**127 N**	**T1(Control)**	**T13(Control)**
**127C**	**T7(Control)**	**T15(Control)**
**128 N**	**T8(Control)**	**T17Control)**
**128C**	**T9(Control)**	**T19(Control)**
**129 N**	**T3(Patient)**	**T11(Patient)**
**129C**	**T4(Patient)**	**T12(Patient)**
**130 N**	**T5(Patient)**	**T14(Patient)**
**130C**	**T6(Patient)**	**T16(Patient)**
**131**	**T10(Patient)**	**T18(Patient)**

To quench the excess TMT label in the sample, 5% hydroxylamine was added to each of the sample and vortexed then incubated at RT for 15 min. Before pooling the samples, to ensure equal amount of total peptides are pooled from all samples, a “label check” experiment was performed by mixing 1.5 μL of each individually labelled TMT sample, vacuum dried. Samples were reconstituted in 2% ACN, 0.1% FA in water and analysed by LC-coupled to a mass spectrometer (Q-Exactive, Thermo Fisher, USA). TMT-labeled peptide samples were pooled at a 1:1 ratio across all samples and vacuum dried after the label check experiment gave a normalisation factor. The samples were vacuum centrifuged to dryness after desalting with C18 solid-phase extraction (SPE, Sep-Pak, Waters). Prior to LC-MS/MS analysis, the peptide mixture was separated into 96 fractions using high pH (HpH) reverse phase HPLC, and then consolidated into 17 fractions. The HpH HPLC fractions from each TMT set were reconstituted using sample loading buffer (2% acetonitrile, 97.9% water, 0.1% formic acid) before LC-MS/MS analysis.

### LC–MS/MS analysis

#### 1D data dependent acquisition (DDA) of peptides on QExactive quadrupole-Orbitrap (QE-classic)

Peptide samples which were TMT labelled were inserted onto an in-house packed trap column and desalted using loading buffer. Peptides were eluted from the trap into an in-house packed analytical column using linear mobile phase A and B gradients: mobile phase B (30%) over 110 minutes at a flow rate of 300 nL/min throughout the gradient.

The eluent was separated from the trap using an analytical column. The eluent from the column was injected into the ionisation source of the mass spectrometer. A 2.6 kV electrospray voltage was applied through a liquid connection upstream of the column. Peptide precursors from 350 to 1850 m/z were scanned at 70 k resolution with an AGC target value of 1 × 106.Higher-Energy Collisional Dissociation (HCD) was used to fragment the 10 most energetic ions from the previous survey scan, with a normalised collision energy of 35 and an isolation width of 0.7 m/z.MS/MS analysis is limited to precursors with a charge state of + 2 to + 4. For MS2 triggering, the MS technique had a minimum signal needed value of 2.5 × 104, an AGC target value of 2105, and a maximum injection duration of 250 ms. The resolution of the MS/MS scan was set at 70 k. The duration of the dynamic exclusion was set to 90 seconds.

### Protein identification and quantification

The mass spectrometric data files for each sample set were searched using Proteome Discoverer (version 2.1, Thermo Scientific). uniprot database (181005_UniPr_HUMAN_Revi+Unrevi.fasta, Ref: http://www.uniprot.org) containing 95,106 human proteins including isoforms and unreviewed (*Homo sapiens*) was used for searching the data. The quantitative ratios were generated using the quantitative values found in channel 126 (control) as denominator.

### Statistical analysis

The differentially abundant proteins were analyzed using the Limma Packages, which are written in R. The Bioconductor software was used to analyze the data, which fits a linear model to each protein and then uses the Bayes technique to the predicted variances to increase power. Comparison of normalized protein areas between control and patient samples (Fig. 2) showed a total of 24 differentially abundant proteins (Fold change 1 ± 0.2, statistically significant differences between patient and control were accepted as *p*-value< 0.05) in tear samples (Fig. 3). The reason for choosing this Fold change was due to the chronic nature of the disease. By selecting a higher threshold, it is difficult to find the altered proteins for patients with chronic disease. Moreover, regarding the limited number of samples, Adaptation of Adjusted *p*-value for a low number of samples limits the validation of significant proteins.

## Results

**Fig. 1:**
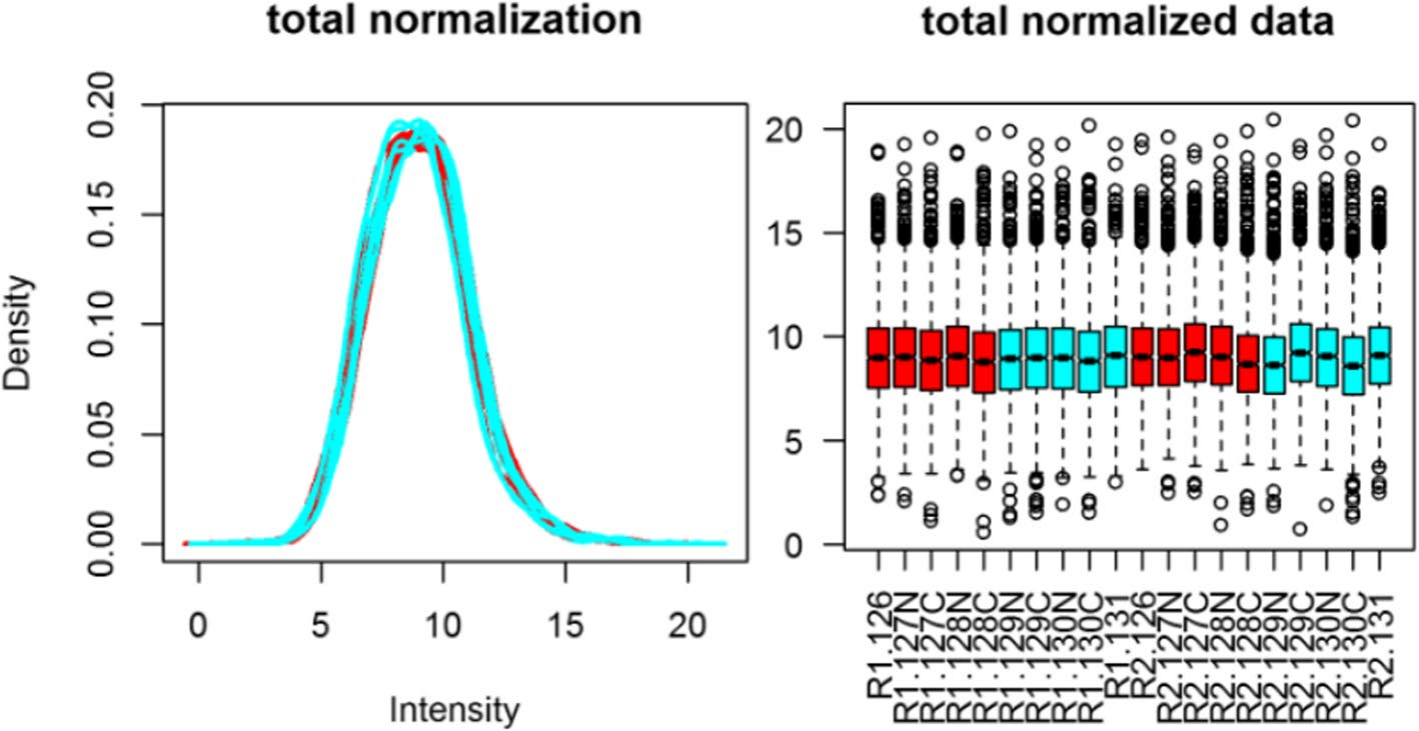
Density plot (left panel) and Box plots (right panel) of post-normalized data showing variability in the TMT-MS data tear

TMT quantitative proteomic analysis of human samples resulted in the identification 3586 high confident (Protein, Peptide and PSM FDR < 1%) proteins in tear samples. A relative abundance comparison was performed using a density plot and Box Plot analysis of normalized data (Fig. 1). Because all samples within each data set have similar protein ratio distributions (ratio with reference to channel 126, a control sample), a relative abundance comparison was performed.

**Fig. 2:**
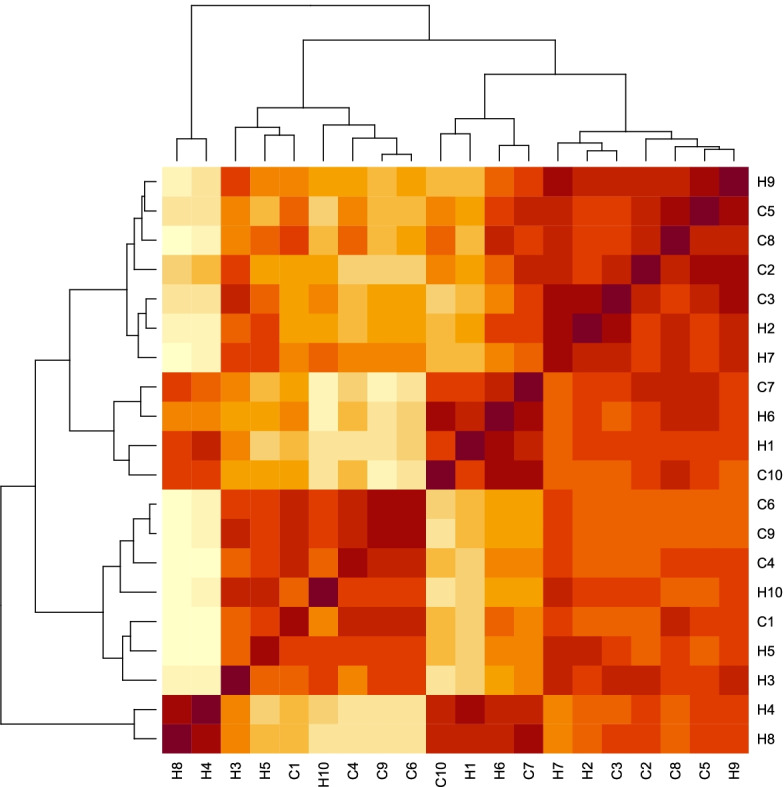
Heatmap plot for visualize hierarchical clustering

One way to pre-process and detect outlier data is to use a heatmap plot that helps visualize hierarchical clustering. Using heatmap package in R, the detection of the outlier sample was performed, which can be seen in Fig. 2. By analyzing the heatmap, it was found that there is no outlier and all samples were analyzed for the next step.

**Fig. 3:**
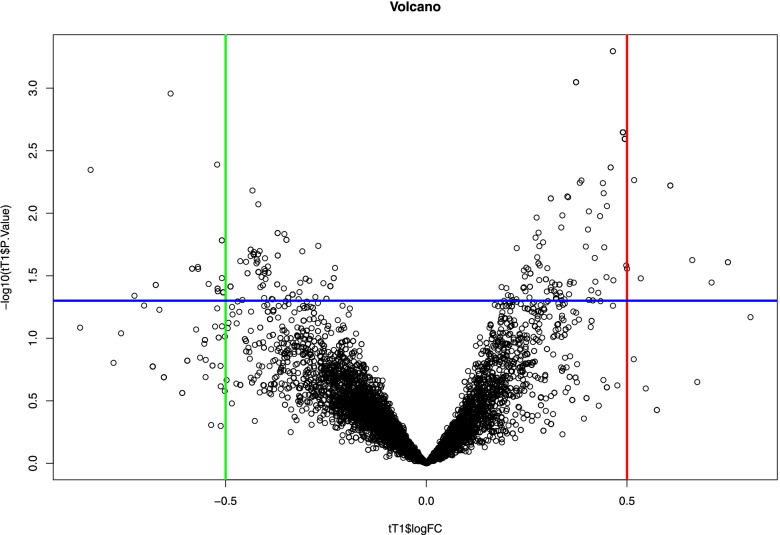
Volcano plot of differentially abundant proteins in the tear

After the data preprocessing and grouping the samples, the list of all differentially abundant proteins was calculated using the Lima package in R language. The final output of this analysis is shown in Fig. 3 as a volcano plot.

The tear protein profiles of 10 healthy individuals and 10 veterans with mustard gas ocular poisoning were compared and our results showed that 24 proteins had different expressions between two groups. As shown in Table 2, of these 24 proteins, 8 proteins had increased expression in veterans’ tears, while the remaining 16 proteins had decreased expression.

**Table 2:** Characteristics of differentially expressed proteins between cases and controls

Accession ID	Protein Name	Gene name	Fold change	*P*-value
Q07654	Trefoil factor 3	TFF3	1.752	0.001
Q8TAX7	Mucin-7	MUC7	1.568	0.001
Q08J23	RNA cytosine C(5)-methyltransferase	NSUN2	1.564	0.006
P52298	Nuclear cap-binding protein subunit 2	NCBP2	1.528	0.011
P05161	Ubiquitin-like protein ISG15	ISG15	1.516	0.004
Q7LGC8	Carbohydrate sulfotransferase 3	CHST3	1.479	0.001
A0A0G2JS65	Mucin-4	MUC4	1.438	0.006
P12273	Prolactin-inducible protein	PIP	1.437	0.044
J3KNN3	Phosphorylase kinase	PHKG2	0.707	0.001
P36957	Dihydrolipoyllysine-residue succinyltransferase component of 2-oxoglutarate dehydrogenase complex, mitochondrial	DLST	0.692	0.022
P06276	Cholinesterase	BCHE	0.689	0.001
Q02318	Sterol 26-hydroxylase, mitochondrial	CYP27A1	0.684	0.021
Q96BM9	ADP-ribosylation factor-like protein 8A	ARL8A	0.681	0.031
Q9NVJ2	ADP-ribosylation factor-like protein 8B	ARL8B	0.681	0.031
Q9BXB5	Oxysterol-binding protein-related protein 10	OSBPL10	0.673	0.036
P08123	Collagen alpha-2(I) chain	COL1A2	0.627	0.030
P04003	C4b-binding protein alpha chain	C4BPA	0.621	0.038
O75636	Ficolin-3	FCN3	0.617	0.003
P16403	Histone H1.2	H1–2	0.616	0.006
H3BSW6	Cytoplasmic tRNA 2-thiolation protein 2	CTU2	0.610	0.001
P20851	C4b-binding protein beta chain	C4BPB	0.574	0.002
P01701	Immunoglobulin lambda variable 1–51	IGLV1–51	0.572	0.006
Q9GZP8	Immortalization up-regulated protein	IMUP	0.458	0.002
A0A075B6I4	Immunoglobulin lambda variable 10–54	IGLV10–54	0.425	0.008

Reactome pathways were used to investigate proteins with various expressions in order to determine the activities and pathways involved (Table 3). Thirteen of the 24 proteins were found to be involved in the immune system, with nine of them being engaged in the innate immune system. In addition, five proteins participated in the complement cascade.

**Table 3:** Processes and pathways that differentially expressed proteins are involved in based on Reactome pathways

Pathway name	Number of entities found	Number of total entities	Proteins involved (labels)
Complement cascade	5	156	FCN3; IGLV1–51; C4BPA; C4BPB; IGLV10–54
Regulation of Complement cascade	4	139	IGLV1–51; C4BPA; C4BPB; IGLV10–54
Innate Immune System	9	1329	FCN3; IGLV1–51; MUC7; C4BPA; ISG15; C4BPB; MUC4; ARL8A; IGLV10–54
Defective GALNT3 causes familial hyperphosphatemic tumoral calcinosis (HFTC)	2	20	MUC7; MUC4
Defective GALNT12 causes colorectal cancer 1 (CRCS1)	2	20	MUC7; MUC4
Defective C1GALT1C1 causes Tn polyagglutination syndrome (TNPS)	2	21	MUC7; MUC4
Creation of C4 and C2 activators	3	111	FCN3; IGLV1–51; IGLV10–54
Termination of O-glycan biosynthesis	2	28	MUC7; MUC4
Initial triggering of complement	3	120	FCN3; IGLV1–51; IGLV10–54
Immune System	13	2869	FCN3; IGLV1–51; COL1A2; MUC7; C4BPA; ISG15; C4BPB; MUC4; ARL8A; IGLV10–54
Binding and Uptake of Ligands by Scavenger Receptors	3	167	IGLV1–51; COL1A2; IGLV10–54
Diseases of glycosylation	3	202	MUC7; MUC4; CHST3
Dectin-2 family	2	65	MUC7; MUC4
Diseases of metabolism	4	409	CYP27A1; MUC7; MUC4; CHST3
tRNA modification in the nucleus and cytosol	2	70	CTU2; NSUN2
O-linked glycosylation of mucins	2	73	MUC7; MUC4
Chondroitin sulfate/dermatan sulfate metabolism	2	73	PIP; CHST3
Diseases associated with O-glycosylation of proteins	2	78	MUC7; MUC4
Defective CYP27A1 causes Cerebrotendinous xanthomatosis (CTX)	1	6	CYP27A1
Cell surface interactions at the vascular wall	3	257	IGLV1–51; COL1A2; IGLV10–54
Classical antibody-mediated complement activation	2	97	IGLV1–51; IGLV10–54
Defective CHST3 causes SEDCJD	1	9	CHST3
FCGR activation	2	103	IGLV1–51; IGLV10–54
Scavenging of heme from plasma	2	106	IGLV1–51; IGLV10–54
Role of LAT2/NTAL/LAB on calcium mobilization	2	107	IGLV1–51; IGLV10–54
SLBP independent Processing of Histone Pre-mRNAs	1	10	NCBP2
SLBP Dependent Processing of Replication-Dependent Histone Pre-mRNAs	1	11	NCBP2
GP1b-IX-V activation signalling	1	12	COL1A2
Ficolins bind to repetitive carbohydrate structures on the target cell surface	1	12	FCN3

Proteins with different expressions were examined using Gene Ontology (GO) to determine in what part of the cell they are located (Table 4). Of these 24 proteins, 11 proteins were extracellular components.

**Table 4:** Cellular components of differentially expressed proteins based on GO

term description	observed gene count	background gene count	Proteins (labels)
extracellular region	11	2505	BCHE, C4BPA, C4BPB, COL1A2, FCN3, IGLV1–51, IGLV10–54, ISG15, MUC4, PIP, TFF3
extracellular region part	8	1375	C4BPA, C4BPB, COL1A2, IGLV1–51, IGLV10–54, MUC4, PIP, TFF3
extracellular space	7	1134	C4BPA, C4BPB, COL1A2, IGLV1–51, IGLV10–54, PIP, TFF
other organism cell	3	36	C4BPA, C4BPB, MUC7
spindle midzone	2	31	ARL8A, ARL8B

## Discussion

The tear protein profiles of 10 veterans with long-term ocular complications due to mustard gas poisoning were compared with the tears of healthy individuals. Our results showed the dysregulated expression of 24 proteins in patients compared to controls, and this could be the key to understanding the pathogenesis of ocular mustard gas poisoning and help find more appropriate treatments for these patients. Thirteen of the 24 proteins with different expressions were involved in immune-related processes, and six proteins were involved in complement cascade function. As a result, it seems that a disturbance in these two biological systems is the primary cause of eye difficulties in mustard gas poisoning patients. TFF3 protein was found to be elevated in our patients’ tears. The expression of this protein is not detectable in normal circumstances and healthy corneas, but it is detectable and enhanced in pathological situations such as Fox dystrophy, herpes keratitis, keratoconus, and pterygium [17, 18]. It was shown that the expression of this protein increases after corneal epithelial damage in mice [19]. This protein binds to mucins and is involved in the packaging and secretion of mucins, thereby altering the flow and concentration of mucus [20]. In the patients with dry eye disease, the expression of this protein increases in terms of corneal damage and similar stressors that cause inflammation [21]. TFF3 is known as the initiator of the wound healing process, which does this by increasing the migration of adjacent epithelial cells. This protein, on the other hand, causes apoptosis and destroys the extracellular matrix in chronic inflammatory situations by activating certain MMPs [17]. Given that increased TFF3 expression activates MMPs, destroys the extracellular matrix, induces apoptosis, and continues the inflammatory process at the corneal surface, it can be concluded that increased TFF3 expression activates MMPs, destroys the extracellular matrix, induces apoptosis, and continues the inflammatory process at the corneal surface. Thus, this vicious cycle of inflammation - increased TFF3 expression - more inflammation can be one of the aspects of ocular mustard gas poisoning pathogenesis.

As mentioned above, TFF3 is involved in the secretion of mucins [20]. In our study, the amount of mucin 4 and 7 was higher in tears of patients than in the control group. However, similar to dry eye patients mucin glycolysis may be reduced in the patients with mustard gas poisoning [22], and increased mucin expression may be a secondary response to this reduction in glycolysis, which requires further research in this area. Mucins play a role in the humoral immune response and signaling process of type C lectin receptors, which are involved in complement activation [23], which may indicate a possible role for these proteins in the pathogenesis of ocular mustard gas poisoning.

PIP protein was significantly upregulated in the tear profile of our patients. The expression of this protein is reduced in patients with dry eye secondary to Sjogren’s disease [24]. Several additional studies showed that people with dry eye had lower levels of this protein in their tears [25–28]. This protein’s expression has also been demonstrated to be reduced in chronic blepharitis [15]. PIP, on the other hand, has been proposed as a Keratoconus biomarker since it is elevated in the tears of keratoconus patients [29]. The effect of PIP on creatine production in the stroma and corneal epithelial cells has also been reported and this indicates the role of this protein in the structural stability of the cornea [30]. Another point about this protein is its role in the immune system; this protein is a specific inhibitor of apoptosis induced by CD4 + T cells and thus has a regulatory role in innate and humoral immunity [31]. Increased expression of this protein was reported in patients with mycotic keratitis, thyroid eye disease and open-angle glaucoma [32–34]. In our study, the expression of PIP protein in the tears of veterans was higher than healthy individuals. According to previous studies and the role of this protein in regulating the immune system, its increased expression indicates impaired efficiency of immune system in responding to pathogens on the surface of patients’ eyes.

Among 24 proteins that had altered expression in this study, 5 proteins had roles in the function of the complement system, so examining the role of this system in eye diseases can be enlightening. The continuous circulation of the complement inside the eye and its role in the eye’s safety status is known [35, 36]. Complement plays an important role in maintaining corneal health [37]. CD46, CD55, and CD59 are membrane bound complement regulators that are expressed in diverse layers of the cornea, though they are more prevalent in the corneal epithelium [38]. This is significant because the cornea’s surface is continually exposed to numerous pathogens, including bacteria like P.aeruginosa, resulting in the complement system’s persistent activation [39]. In fact, some bacteria produce phospholipases and other enzymes that can remove CD55 and CD59 from the corneal epithelial surface, potentially predisposing the cornea to irregular activation of complement, which exacerbates bacterial keratitis (inflammation of the cornea) ultimately leading to vision loss [39, 40]. In general, just as the continuous function of the complement in the eye is good for maintaining corneal health, too much activity can cause uncontrolled inflammation and damage to the cornea. Therefore, balanced regulation of complement activity is required in the cornea to prevent damage.

According to the results of our study, the tears of patients with ocular mustard gas poisoning had lower levels of complement activating proteins (C4BPA, C4BPB, FCN3, IGLV10–54, IGLV1–51), so it seems that mustard gas pathogenesis can be linked to the reduced activity of complement system in these patients making the cornea more susceptible to pathogens, such as Pseudomonas and external toxic agents.

## Conclusion

We found numerous dysregulated proteins in the tear profiles of patients with sulfur mustard poisoning, and analysis of their activities revealed that these proteins were most typically connected with the innate immune and complement pathways. As a result, it’s possible that abnormalities of the complement system and the innate immune system are involved in the pathogenesis of mustard gas ocular poisoning. In summary, mustard gas in the eye may lead to a weakened immune system on the surface of the eye, predisposing the cornea to pathogens, and ultimately causing corneal opacity and decreased vision. To confirmation and validation of this data, more experimentally research should be conducted on the complement system and innate immunity activity in the eye of these patients. After validation and confirmation of these finding in future studies, new drugs or protocols can be proposed for treatment of delayed mustard gas keratopathy.

## Data Availability

The data that support the findings of this study are available from the corresponding author. upon reasonable request.

## Abbreviations

CWC: Chemical Weapons Convention
OPCW: Organization for the Prohibition of Chemical Weapons
TMT: Tandem Mass Tag
DDA: Data Dependent Acquisition
HCD: Higher-Energy Collisional Dissociation
